# Multiple machine-learning-driven metabolic frameworks for long-term prognostic risk assessment in patients with coexisting hypertension and obstructive sleep apnea:insights from a multicenter cohort study

**DOI:** 10.3389/fphys.2026.1739374

**Published:** 2026-02-24

**Authors:** Qiong Xu, Yanan Xu, Yijun Wang, Shuo Liu, Yi Yang, Hongchang Zhao, Shoupeng Duan, Jun Wang

**Affiliations:** 1 Department of Cardiology, The First Affiliated Hospital of Bengbu Medical University, Bengbu, Anhui, China; 2 Pulmonary and Critical Care Medicine, The First Affiliated Hospital of Bengbu Medical University, Bengbu, Anhui, China; 3 Department of Cardiology, People’s Hospital of Xuancheng City, The Affiliated Xuancheng Hospital of Wannan Medical College, Wuhan, Anhui, China; 4 Center of Gerontology and Geriatrics, National Clinical Research Center for Geriatrics, West China Hospital, Sichuan University, Chengdu, China; 5 First Clinical College, Anhui Medical University, Hefei, China; 6 First Clinical College, Xinjiang Medical University, Urumqi, China; 7 Department of Cardiology Fourth Ward, The Xinjiang Medical University Affiliated Hospital of Traditional Chinese Medicine, Urumqi, China; 8 Department of Emergency Surgery, The First Affiliated Hospital of Bengbu Medical University, Bengbu, China; 9 Department of Cardiology, Renmin Hospital of Wuhan University, Wuhan, China; 10 Joint Research Center for Regional Diseases of IHM, The First Affiliated Hospital of Bengbu Medical University, Bengbu, Anhui, China

**Keywords:** hypertension, machine-learning, metabolic framework, obesity, obstructive sleep apnea

## Abstract

**Background:**

Predictive obesity indices are often based on the body mass index (BMI). Although BMI is widely used, it does not provide a direct measure of obesity. We aimed to utilize multiple machine learning-driven metabolic frameworks to investigate the long-term risk of major adverse cardiovascular and cerebrovascular events (MACCEs) in individuals with hypertension and obstructive sleep apnea (OSA).

**Methods:**

This study included 708 patients with hypertension and OSA between January 2017 and December 2021. The measurements of height, weight, neck circumference (NC), waist circumference (WC), neck-circumference-to-height ratio (NHtR), and waist-to-height ratio (WHtR) were collected to calculate the triglyceride-glucose (TyG)-BMI, as well as TyG-NC, TyG-WC, TyG-NHtR, and TyG-WHtR indices.

**Results:**

All patients were allocated to the training cohort (n = 446) and independent validation cohort (n = 262). The Boruta plot presented for identifying key predictors is as follows: male sex, age, TyG, TyG-BMI, HbA1c, FPG, triglyceride, creatinine, fibrinogen and AHI. We constructed nine machine learning models-XGBoost, Light Gradient Boosting Machine, Random Forest, Decision Tree, Gradient Boosting, Multi-Layer Perceptron, Support Vector Machine, K-Nearest Neighbors, and Gaussian Naive Bayes-to predict MACCEs. The XGBoost model was selected due to its superior performance evidenced by an AUC of 0.898 (95% CI: 0.822–0.973) and net clinical benefit. SHAP analysis further clarified variable contributions to MACCE risk.

**Conclusion:**

This study employed various machine-learning techniques and multidimensional data assessment, allowing for enhanced prediction of metabolic results and supporting the timely detection of high-risk patients with OSA and hypertension in need of focused preventive measures.

**Clinical Trial Registration:**

https://www.chictr.org.cn/bin/project/edit?pid=206415, identifier ChiCTR2300075727.

## Introduction

Obesity, a major global public health challenge, is strongly associated with several metabolic disorders, such as insulin resistance (IR), type 2 diabetes mellitus, and cardiovascular disease (Czech, 2017; Tian et al., 2022; Powell-Wiley et al., 2021). Traditionally, obesity assessment has relied primarily on body mass index (BMI), which is weight (in kilograms) divided by height squared (in meters). Despite its widespread clinical use, BMI does not reflect how specific distributions of adipose tissue affect obesity severity (Prillaman, 2023). Therefore, over time, researchers have realized the limitations associated with relying solely on BMI to assess obesity and have attempted to redefine obesity in terms of multidimensional indicators, which include not only BMI but also neck circumference (NC), waist circumference (WC), NC-to-height ratio (NHtR), and WC-to-height ratio (WHtR) (Lv et al., 2024; Cai et al., 2023; Busetto et al., 2024; Zhao et al., 2024; Ren et al., 2024). These indicators can more accurately reflect the potential health effects of different fat distribution.

Notably, obstructive sleep apnea (OSA) and hypertension are two common diseases that are closely related to and often coexist with obesity and aggravate IR and metabolic disorders in patients (Aminian et al., 2024; Zhou et al., 2024). Patients with OSA often experience intermittent hypoxemia and decreased sleep quality (Aminian et al., 2024; Zhou et al., 2024; Xu et al., 2022; Xu et al., 2024; Yeghiazarians et al., 2021). Moreover, repeated apnea and hypopnea can lead to sympathetic hyperactivity, which leads to blood pressure fluctuation and increased challenges with blood pressure control (Yeghiazarians et al., 2021; He et al., 2020). Therefore, the comorbidity of OSA and hypertension is not limited to a simple concurrent relationship but involves the interaction of various complex physiological and pathological pathways that jointly aggravate the occurrence and development of cardiovascular disease (Aminian et al., 2024; Zhou et al., 2024; Xu et al., 2022; Xu et al., 2024; Yeghiazarians et al., 2021; He et al., 2020; Floras, 2015; Javaheri et al., 2017).

Notably, a growing body of research evidence demonstrates that machine learning can effectively elucidate the association between the aggregation of multiple disease factors and long-term risks (Johnson et al., 2018; Ye et al., 2023; Wang J. et al., 2025). This capability facilitates a more nuanced analysis of how various risk factors interact and contribute to disease progression, thereby enhancing our ability to predict outcomes and tailor interventions for patients (Johnson et al., 2018; Ye et al., 2023; Wang J. et al., 2025). By leveraging machine learning techniques, we can uncover intricate patterns within large datasets, leading to improved risk stratification and more personalized approaches to disease management (Johnson et al., 2018; Chen et al., 2024).

While recent studies have demonstrated the superiority of alternative obesity indices and metabolic biomarkers individually, there remains a critical gap in the development of comprehensive risk stratification frameworks specifically designed for OSA-hypertension populations (Cai et al., 2023; Zhao et al., 2024; Aminian et al., 2024). Despite the documented limitations of traditional risk assessment tools and the emerging evidence supporting novel metabolic indices, limited investigation has been conducted into their integration with sophisticated computational methodologies for enhanced predictive accuracy. This knowledge gap represents a significant barrier to optimal clinical decision-making and personalized therapeutic interventions in this high-risk patient population. Therefore, to address the inherent limitations of conventional BMI-based obesity assessment and bridge this critical knowledge gap, this study aimed to develop and validate a comprehensive risk stratification framework. We hypothesized that effective cardiovascular risk prediction in patients with OSA and hypertension requires an integrated evaluation of metabolic, anthropometric, demographic, and clinical parameters, rather than reliance on any single measurement. This approach addresses the dual challenge of metabolic complexity and predictive accuracy by leveraging both multiple types of data and multiple machine learning methodologies.

## Methods

### Study design and participants

**FIGURE 1:**
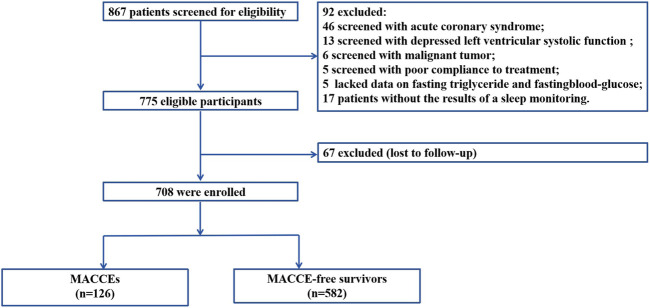
Flowchart of the inclusion of participants in the study.

This multicenter cohort study enrolled 708 patients with OSA and hypertension who were hospitalized in the Xinjiang Medical University Affiliated Hospital of Traditional Chinese Medicine from January 2017 to December 2019 and People’s Hospital of Xuancheng City from January 2018 to August 2021 (Figure 1). Inclusion and exclusion criteria with detailed, objective definitions in Supplementary Table 1. This study employed a multicenter cohort design utilizing clinical data from two geographically distinct institutions in China: the training cohort (n = 446) was derived from the Xinjiang Medical University Affiliated Hospital of Traditional Chinese Medicine, while the independent validation cohort (n = 262) was recruited from the People’s Hospital of Xuancheng City. The study adhered to the tenets of the Declaration of Helsinki and was conducted in accordance with the regulations of our hospital. All participants underwent overnight polysomnography (PSG) during hospitalization and experienced clinically defined OSA according to the American Academy of Sleep Medicine guidelines; their severity of OSA was assessed according to the apnea-hypopnea index (AHI) (Kapur et al., 2017).

### Anthropometric measurement protocols

All anthropometric measurements were performed by trained clinical staff following standardized protocols to ensure consistency and accuracy. Height was measured using a wall-mounted stadiometer with patients standing barefoot in an upright position, recorded to the nearest 0.1 cm. Weight was obtained using calibrated digital scales with patients wearing light clothing, recorded to the nearest 0.1 kg. Body Mass Index (BMI) was calculated as weight (kg) divided by height squared (m^2^). Neck circumference was measured at the level of the cricothyroid membrane using a flexible tape measure while patients were seated in a neutral head position, ensuring the tape was snug but not compressing the soft tissues, and recorded to the nearest 0.1 cm. Waist circumference was measured at the midpoint between the lowest rib and the iliac crest during normal expiration, recorded to the nearest 0.1 cm.

### TyG index and modified TyG indices

In this study, the triglyceride-glucose (TyG) index was calculated by obtaining fasting blood specimens from all participants after they had undergone a minimum 8-h overnight fast on the first day of hospital admission. This procedure was conducted to measure their blood glucose and triglyceride levels. Height, NC, and WC were measured three times, and the average of the three measurements was considered as the final value. The TyG index was calculated as the natural logarithm of fasting serum triglycerides (mg/dL) multiplied by fasting blood glucose (FBG) (mg/dL) divided by 2 (Johnson et al., 2018). The body mass index (BMI) was calculated as weight divided by height squared (kg/m^2^); NHtR was defined as NC/height, and WHtR was defined as WC/height. Subsequently, to refine the TyG index, these were multiplied with BMI, NC, WC, NHtR, and WHtR, resulting in TyG-BMI, TyG-NC, TyG-WC, TyG-NHtR, and TyG-WHtR indices, respectively (Cui et al., 2024).

### Follow-up and clinical endpoint

The primary clinical endpoint was Major Adverse Cardiovascular and Cerebrovascular Events (MACCEs), which encompassed cardiac mortality, acute coronary syndrome, and stroke. Cardiac mortality included death due to myocardial infarction, heart failure, arrhythmias, or sudden cardiac death. Acute coronary syndrome was defined according to contemporary guidelines, including unstable angina, non-ST-elevation myocardial infarction, and ST-elevation myocardial infarction, diagnosed based on clinical presentation, electrocardiographic changes, and cardiac biomarker elevations. Stroke was defined as acute onset of focal neurological deficit lasting >24 h with confirmatory neuroimaging evidence of cerebral infarction or hemorrhage.

Clinical follow-up was conducted by dedicated physicians who were blinded to the patients’ baseline risk stratification and predictive model classifications. Follow-up involved standardized questionnaires and relevant clinical examinations performed telephonically or during scheduled outpatient visits. All potential MACCE events were systematically documented and verified through comprehensive medical record review and independent physician adjudication to ensure objective and unbiased outcome assessment.

Patients were followed from the date of initial OSA diagnosis until the occurrence of a MACCE, death from non-cardiovascular causes, loss to follow-up, or study termination, whichever occurred first. The median follow-up duration was 47.13 months. Patients who experienced non-cardiovascular death were censored at the time of death. Patients lost to follow-up were censored at the date of last known clinical contact. The systematic follow-up protocol, conducted by specialized clinical staff, ensured comprehensive and objective ascertainment of clinical outcomes while maintaining data quality and completeness throughout the observation period.

### Handling missing data

As part of our predefined exclusion criteria, patients with missing values for any variables included in the analysis were systematically excluded from the study. This complete case analysis strategy ensured absolute data integrity across the entire analytical cohort and was methodologically justified by several considerations. By including only cases with fully observed data, we avoided potential biases associated with imputation methods, ensuring that all analyses were based on actual, verified clinical measurements rather than estimated values.

### Multiple machine-learning-driven prediction model

To identify significant predictors of MACCEs, we applied the Boruta algorithm, an advanced feature selection method that systematically eliminates irrelevant features while retaining those deemed important. The variance inflation factor (VIF) was calculated to assess potential multicollinearity. This sequential analytical approach ensured methodologically rigorous variable selection while preserving model interpretability, which is essential for clinical application (Johnston, 1984). Variables with VIF values below 10 suggest that the predictors retain sufficient unique variance and do not exhibit problematic intercorrelation that would compromise model stability or coefficient interpretability (Johnston, 1984). Conversely, VIF values exceeding 10 would indicate severe multicollinearity concerns and suggest poorly constructed model specifications requiring variable elimination or transformation (Johnston, 1984).

Following this, we employed nine distinct machine learning algorithms for classification and prediction of MACCEs. These machine learning algorithms included the eXtreme Gradient Boosting (XGBoost), Light Gradient Boosting Machine (LightGBM), Random Forest, Decision Tree, Gradient Boosting, Multi-Layer Perceptron, Support Vector Machine (SVM), K-Nearest Neighbors (KNN), and Gaussian Naive Bayes (GNB). To contextualize the performance of these machine learning models within established statistical frameworks, we additionally constructed a Cox proportional hazards regression model utilizing backward stepwise selection as a benchmark comparator. This dual analytical approach enabled a comprehensive evaluation of both the independent predictive value of individual risk factors and the incremental discriminative advantage conferred by advanced machine learning algorithms over conventional regression-based methods. Each model was trained on the training cohort, with hyperparameter optimization performed via grid search to improve predictive performance. The performance of each machine learning model was evaluated using a comprehensive set of metrics in both the training and independent validation cohorts, including the area under the receiver operating characteristic curve (AUC), accuracy, sensitivity, specificity, positive predictive value, negative predictive value, F1-score, Cohen’s kappa coefficient, and Brier score. These metrics assess the model’s ability to discriminate between positive and negative outcomes. Calibration Curves were used to evaluate the concordance between predicted probabilities and observed outcomes. Decision Curve Analysis (DCA) was conducted to examine the clinical utility of the models by comparing net benefits across different decision thresholds. Furthermore, SHAP (SHapley Additive exPlanations) values were employed to provide insights into the contributions of individual features to the model’s predictions. SHAP values offer a consistent measure of feature importance and facilitate the visualization of each feature’s influence on the predicted outcome.

### Statistical analysis

Statistical analysis was conducted using python version 3.11.4 and R software 4.2.2 (R Core Team), and *P* < 0.05 was considered statistically significant. Categorical variables were expressed as frequencies and percentages, whereas continuous variables were expressed as medians with quartiles or means with standard deviations. Baseline characteristics of the training and independent validation cohorts were compared using the Fisher exact test or χ^2^ test for categorical variables and the Mann-Whitney U test for non-normally distributed continuous variables.

## Results

### Patient characteristics

This multicenter cohort study included 708 patients having OSA with hypertension, with a median follow-up of 47.13 months. This study employed a multicenter cohort study design and utilized clinical data provided by the institution to stratify patients into two cohorts: the training cohort (n = 446) from the Xinjiang Medical University Affiliated Hospital of Traditional Chinese Medicine and the independent validation cohort (n = 262) from the People’s Hospital of Xuancheng City. The training and independent validation cohorts were well matched, showing no significant differences between the groups for any baseline measures, except for male sex, high-density lipoprotein cholesterol, and low-density lipoprotein cholesterol (Table 1).

**TABLE 1:** Comparison of patient characteristics between the training and validation cohorts.

Characteristic of outcome	All cohort N = 708	Training cohort N = 446	Validation cohort N = 262	P-overall
Male sex, n (%)	486 (68.64%)	291 (65.25%)	195 (74.43%)	0.014
Age, years	54.00 [47.00; 61.00]	54.00 [47.00; 60.75]	54.00 [47.00; 62.00]	0.776
Diabetes mellitus, n (%)	135 (19.07%)	81 (18.16%)	54 (20.61%)	0.483
Current smoking, n (%)	302 (42.66%)	184 (41.26%)	118 (45.04%)	0.366
Current alcohol consumption, n (%)	273 (38.56%)	163 (36.55%)	110 (41.98%)	0.175
Previous CAD, n (%)	355 (50.14%)	222 (49.78%)	133 (50.76%)	0.860
Family history of CAD, n (%)	113 (15.96%)	74 (16.59%)	39 (14.89%)	0.622
Non alcoholic fatty liver disease, n (%)	389 (54.94%)	245 (54.93%)	144 (54.96%)	1.000
Height, cm	170.00 [164.00; 175.00]	170.00 [164.00; 175.00]	170.00 [164.00; 174.00]	0.218
Weight, cm	81.00 [72.00; 90.00]	82.00 [73.00; 90.00]	80.00 [71.25; 89.00]	0.058
BMI, kg/m^2^	27.89 [25.72; 31.14]	28.29 [25.96; 31.16]	27.61 [25.52; 31.11]	0.097
Waistline, cm	94.00 [88.00; 101.25]	94.00 [88.00; 102.00]	94.00 [87.25; 100.75]	0.876
Waistline/Height	0.56 [0.52; 0.60]	0.55 [0.52; 0.60]	0.56 [0.51; 0.60]	0.689
Neck circumference/Height	0.24 [0.22; 0.26]	0.24 [0.22; 0.26]	0.24 [0.22; 0.26]	0.062
Neck circumference, cm	40.00 [38.00; 43.00]	40.00 [38.00; 43.00]	41.00 [38.00; 44.00]	0.071
TyG	8.92 [8.56; 9.33]	8.93 [8.57; 9.31]	8.90 [8.51; 9.35]	0.570
TyG-BMI	251.61 [227.47; 280.00]	253.26 [231.49; 283.16]	245.40 [222.66; 275.29]	0.071
TyG-neck circumference	360.35 [331.04; 396.59]	357.82 [329.43; 394.21]	363.95 [331.94; 404.75]	0.248
TyG-waistline	842.81 [765.64; 920.42]	842.91 [765.60; 918.63]	841.23 [766.63; 925.02]	0.907
TyG-WHtR	4.97 [4.56; 5.46]	4.95 [4.58; 5.41]	4.97 [4.56; 5.50]	0.741
TyG-neck circumference/Height	2.14 [1.95; 2.37]	2.13 [1.94; 2.35]	2.19 [1.98; 2.41]	0.171
HbA1c,%	5.82 [5.50; 6.40]	5.81 [5.53; 6.36]	5.82 [5.45; 6.49]	0.599
HDL-C, mmol/L	1.23 [1.08; 1.39]	1.24 [1.09; 1.41]	1.21 [1.06; 1.35]	0.017
LDL-C, mmol/L	2.30 [1.83; 2.76]	2.33 [1.87; 2.82]	2.22 [1.73; 2.73]	0.030
Total cholesterol, mmol/L	4.32 [3.65; 4.99]	4.42 [3.76; 5.11]	4.20 [3.44; 4.85]	0.011
Fasting blood glucose, mmol/L	5.27 [4.74; 6.21]	5.24 [4.73; 6.21]	5.28 [4.76; 6.16]	0.944
Triglyceride, mmol/L	1.68 [1.23; 2.36]	1.71 [1.25; 2.37]	1.65 [1.23; 2.32]	0.692
Apolipoprotein A1, g/L	1.22 [1.12; 1.36]	1.22 [1.13; 1.36]	1.21 [1.10; 1.36]	0.368
Apolipoprotein B, g/L	0.92 [0.78; 1.08]	0.93 [0.80; 1.09]	0.92 [0.74; 1.07]	0.079
Lipoprotein(a), mg/L	128.33 [65.93; 261.93]	127.08 [65.14; 269.00]	132.15 [67.36; 249.03]	0.862
Creatinine, μmol/L	76.00 [65.07; 86.00]	75.50 [65.00; 86.97]	77.00 [65.40; 86.00]	0.593
Urea nitrogen, mmol/dL	5.30 [4.60; 6.20]	5.30 [4.60; 6.20]	5.30 [4.50; 6.25]	0.421
Uric acid, μmol/L	346.00 [294.60; 399.57]	343.25 [293.22; 402.57]	347.80 [295.10; 394.60]	0.862
D-dimer, mg/L	0.50 [0.34; 0.64]	0.50 [0.34; 0.64]	0.49 [0.32; 0.65]	0.876
Fibrinogen, g/L	2.94 [2.57; 3.41]	2.98 [2.60; 3.41]	2.86 [2.52; 3.40]	0.332
Hemoglobin, g/L	149.00 [136.00; 158.00]	149.00 [136.00; 157.00]	149.00 [137.00; 159.00]	0.339
White blood cell count, 10^9^/L	6.53 [5.53; 7.61]	6.50 [5.50; 7.64]	6.54 [5.60; 7.57]	0.991
Neutrophil count, 10^9^/L	4.22 [3.19; 5.62]	4.20 [3.17; 5.70]	4.23 [3.25; 5.56]	0.849
Lymphocyte count, 10^9^/L	2.26 [1.81; 2.84]	2.28 [1.80; 2.86]	2.21 [1.82; 2.81]	0.438
Neutrophil count/Lymphocyte count	1.84 [1.48; 2.39]	1.83 [1.46; 2.39]	1.88 [1.51; 2.39]	0.400
Platelet count, 10^9^/L	235.00 [197.00; 272.00]	235.00 [196.25; 275.75]	234.50 [197.25; 269.00]	0.732
Platelet count/Lymphocyte count	104.25 [80.98; 132.97]	103.51 [79.45; 132.40]	106.08 [85.46; 133.76]	0.460
Platelet distribution width, %	11.90 [10.80; 13.30]	11.85 [10.80; 13.20]	11.90 [11.00; 13.60]	0.128
Mean platelet volume, fL	10.20 [9.70; 10.90]	10.20 [9.60; 10.90]	10.25 [9.70; 11.00]	0.235
AHI	16.20 [9.67; 29.33]	16.00 [9.80; 28.88]	16.60 [9.50; 29.85]	0.831
MACCEs, n (%)	126 (17.80%)	73 (16.37%)	53 (20.23%)	0.232
Cardiac mortality, n (%)	17 (2.40%)	11 (2.47%)	6 (2.29%)	1.000
Acute coronary syndrome, n (%)	105 (14.83%)	58 (13.00%)	47 (17.94%)	0.094
Stroke, n (%)	22 (3.11%)	15 (3.36%)	7 (2.67%)	0.774

* Values are given as n (%), mean ± SD, or median [IQR].

Abbreviations: CAD, coronary artery disease; BMI, body mass index; WHtR, WC-to-height ratio; HDL-C, high-density lipoprotein cholesterol; LDL-C, low-density lipoprotein cholesterol; TyG index, triglyceride-glucose index; MACCEs, main adverse cardiovascular events; AHI, apnea-hypopnea index.

### Multiple machine-learning-driven metabolic frameworks

**FIGURE 2:**
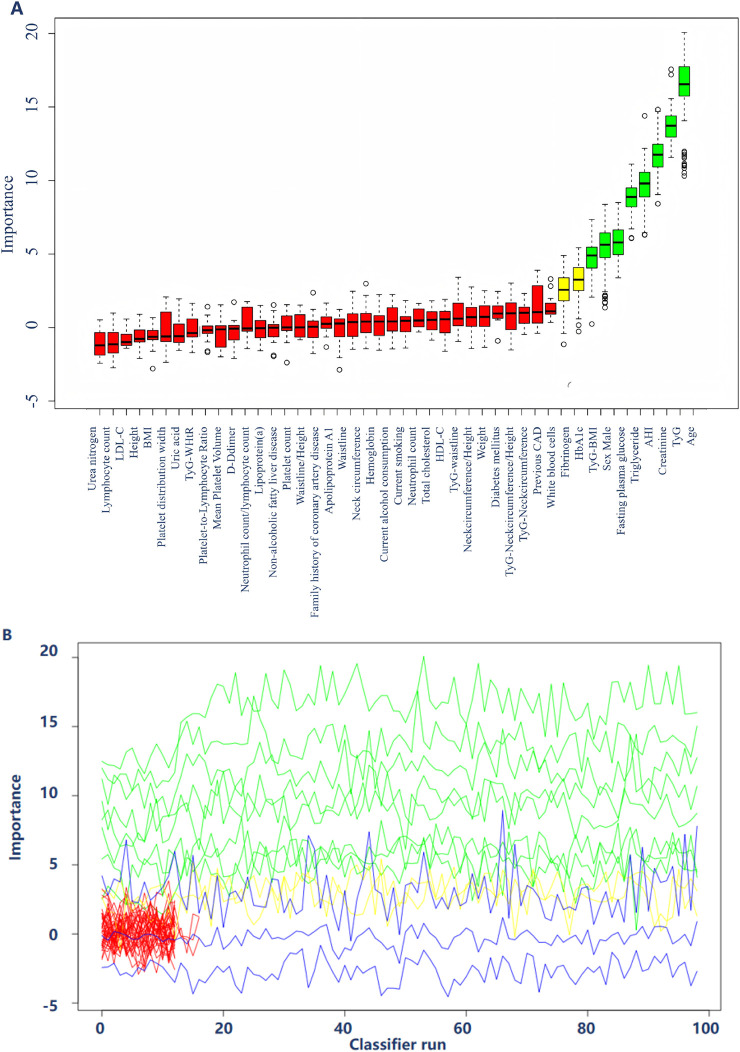
The Boruta plot illustrates the significance of diverse clinical and metabolic indicators in forecasting major adverse cardiovascular and cerebrovascular events (MACCEs) associated with concurrent hypertension and obstructive sleep apnea. The x-axis represents each variable, while the y-axis indicates the importance score, reflecting the contribution of each factor to the predictive model **(A)**. **(B)** Depicts the significance of various predictors across multiple classifier iterations, with the y-axis denoting “Importance” and the x-axis representing “Classifier Run.” The lines are color-coded to reflect different levels of importance: green lines denote predictors of high importance, yellow lines indicate predictors of moderate importance, and blue lines signify predictors of lower importance.

In the training cohort, the Boruta plot presented in this visualization highlights the multifactorial nature of cardiovascular risk assessment and demonstrates the potential utility of machine learning in identifying key predictors within a diverse array of clinical parameters (Figures 2A,B). The following variables were selected as significant predictors: male sex, age, TyG, TyG-BMI, HbA1c, FPG, triglyceride, creatinine, fibrinogen and AHI. According to the VIF analysis, all variables demonstrated VIF values substantially below the conventional threshold of 10, indicating the absence of significant multicollinearity issues (Supplementary Table 2).

We constructed nine machine learning models-XGBoost, LightGBM, Random Forest, Decision Tree, Gradient Boosting, Multi-Layer Perceptron, SVM, KNN, and GNB-to predict the long-term prognosis of patients with coexisting OSA and hypertension. After hyperparameter optimization, these models were trained on the entire training cohort and their performance was subsequently evaluated using the independent validation cohort (Supplementary Table 3).

**FIGURE 3:**
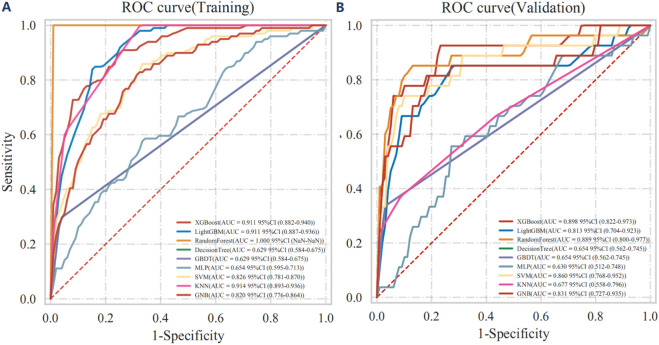
The Receiver Operating Characteristic (ROC) curves for both the training **(A)** and independent validation **(B)** cohorts are presented, demonstrating the performance of various machine learning classifiers in outcome prediction.

The ROC curve analysis of the training cohort revealed that XGBoost and LightGBM achieved the highest AUC values, indicative of superior predictive performance (Figure 3A and Supplementary Figure 1A). In the independent validation cohort, the AUC values for each model were as follows: XGBoost: AUC = 0.898 (95% CI: 0.822–0.973); LightGBM: AUC = 0.813 (95% CI: 0.704–0.923); Random Forest: AUC = 0.889 (95% CI: 0.800–0.977); Decision Tree: AUC = 0.654 (95% CI: 0.562–0.745); GBDT: AUC = 0.654 (95% CI: 0.562–0.745); Multi-Layer Perceptron: AUC = 0.630 (95% CI: 0.512–0.748); SVM: AUC = 0.860 (95% CI: 0.768–0.952); KNN: AUC = 0.677 (95% CI: 0.558–0.796); GNB: AUC = 0.831 (95% CI: 0.727–0.935) (Figure 3B) and Cox proportional hazards regression model with backward stepwise selection: AUC = 0.864 (95% CI: 0.804–0.925) (Supplementary Figure 1B).

**FIGURE 4:**
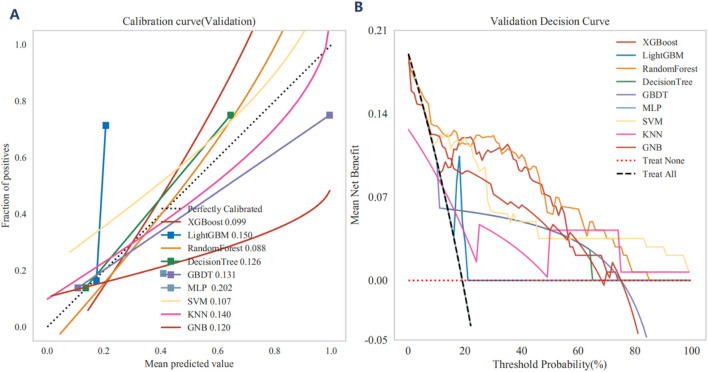
**(A)** The calibration curves for various machine learning, assessed on the independent validation cohort, are presented, demonstrating the relationship between the mean predicted probabilities and the actual fraction of positive outcomes. **(B)** The validation Decision Curve Analysis (DCA) for various machine learning classifiers demonstrates the mean net benefit across a spectrum of threshold probabilities. The x-axis delineates the threshold probability for classifying a positive outcome, whereas the y-axis represents the mean net benefit, thereby indicating the clinical utility of each model in decision-making processes.

**FIGURE 5:**
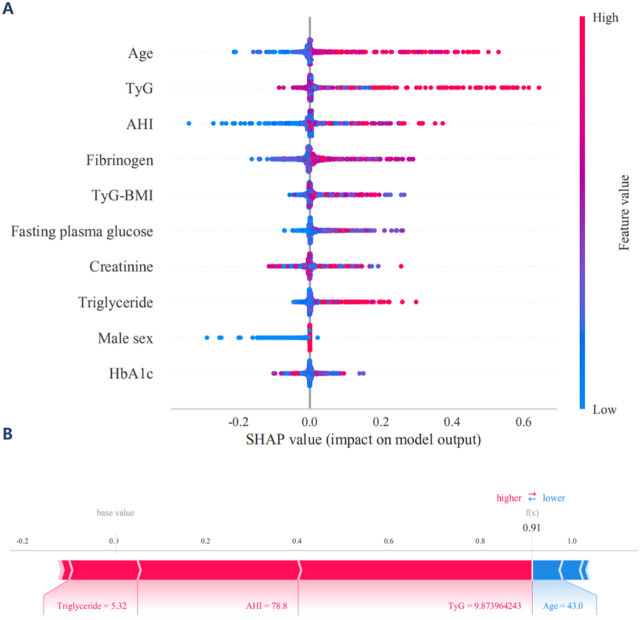
**(A)** SHAP interpretation of the XGBoost model. The SHAP (SHapley Additive exPlanations) summary plot provides a visual representation of the influence exerted by different features on the model’s output. In this plot, the features are arranged along the y-axis, whereas the x-axis denotes the SHAP values, which quantify the contribution of each feature to the prediction. **(B)** The contributions of various clinical and metabolic features to the XGBoost model’s predictions via a waterfall plot. Each panel highlights how individual features influence the predicted outcome relative to a baseline value.

The ROC curve, accuracy, sensitivity, specificity, positive predictive value, negative predictive value, F1-score and Kappa for the independent validation cohort collectively demonstrated that the XGBoost model maintained robust predictive performance (Supplementary Tables 3, 4). Each calibration curve corresponds to a different machine learning, with XGBoost and LightGBM demonstrating the closest alignment to the perfectly calibrated line, indicating superior predictive performance. XGBoost achieves a lowest Brier score of 0.099, while LightGBM has a mean Brier score of 0.150, both accompanied by their respective 95% confidence intervals (Figure 4A). Thus, the calibration curves highlight the effectiveness of each model in predicting MACCEs, with XGBoost and LightGBM demonstrating superior calibration compared to the other machine learning. The DCA highlight the comparative effectiveness of each classifier in guiding clinical decisions, with XGBoost emerging as the most advantageous models for maximizing net benefit in the independent validation cohort (Figure 4B). The SHAP values of XGBoost model provide valuable insights into the relative importance of each feature in predicting MACCEs, highlighting the complex interplay between clinical and metabolic factors (Figure 5A). Notably, age and TyG index emerge as the most influential predictors, with the highest mean absolute SHAP values of XGBoost model, indicating their significant role in the model’s decision-making process (Supplementary Figure 2). The visualization underscores the significance of TyG and TyG-BMI as critical predictors, while also revealing the nuanced contributions of other variables (Supplementary Figure 3). The contributions of various clinical and metabolic features to the model’s predictions regarding MACCEs using SHAP values (Figure 5B). The SHAP values offer valuable insights into the influence of each feature on risk assessment, with TyG, TyG-BMI, and multidimensional clinical data emerging as significant predictors.

## Discussion

This paradigm shift, driven by integrating medical insights with machine learning methodologies, stems from recognizing that BMI alone provides an inadequate diagnostic criterion for obesity-related health risks. Incorporating multidimensional data into predictive models enables more precise evaluation of individual health risks, facilitating the development of personalized prevention and treatment strategies tailored to specific metabolic profiles. This approach enhances our understanding of obesity and its associated health consequences while ultimately improving patient outcomes.

As a global health problem, obesity is not merely a simple consequence of being overweight but also a manifestation of chronic metabolic disorders, which are closely related to IR (Czech, 2017). Notably, current BMI-based measures of obesity may both underestimate and overestimate adiposity, thereby providing insufficiently nuanced information about health at the individual level (Busetto et al., 2024). The increase in adipose tissue mass in people with obesity is not limited to its function as an energy reservoir but involves a range of related endocrine and metabolic activities (Ghaben and Scherer, 2019). Moreover, central obesity, which is an excess of abdominal fat, is considered to be a key trigger of IR (Czech, 2017). Adipocytes in people with obesity can release large amounts of adipokines, which in turn interfere with the insulin signaling pathway, leading to the progression of IR (Czech, 2017). Additionally, obesity is often accompanied by systemic chronic inflammatory response, with inflammatory factors like tumor necrosis factor-α and interleukin-6 exacerbating IR and promoting cardiovascular disease (Liu et al., 2020). To better assess IR, the TyG index has been widely used to reflect triglyceride and FBG metabolism, and it provides a simpler and more accurate assessment tool for IR (Simental-Mendía et al., 2008; Muniyappa et al., 2008; Mohd Nor et al., 2016). However, the current TyG index calculation formula fails to fully capture the role of obesity in IR and cardiovascular disease. Although traditional obesity indicators like BMI are simple and easy to use, they do not factor in the distribution of fat in different parts of the body (e.g., the neck and waist), which differently affects the metabolism and cardiovascular diseases (Er et al., 2016). Therefore, effective assessment of IR and redefining obesity are crucial for reducing cardiovascular disease risk.

Importantly, insulin resistance severity is significantly influenced by adipose tissue distribution and total adiposity load (Pulsford et al., 2025; Packer et al., 2025). Visceral adipose tissue, in particular, contributes to systemic insulin resistance through multiple mechanisms including increased free fatty acid release, pro-inflammatory cytokine secretion, and decreased adiponectin production (Pulsford et al., 2025; Packer et al., 2025). Based on this pathophysiological understanding, we introduced modified TyG indices in our study, including TyG-BMI, TyG-NC, TyG-WC, TyG-NHtR, and TyG-WHtR indices, to more comprehensively evaluate the metabolic risk profile in OSA patients with hypertension. By multiplying TyG by anthropometric indices, these composite markers capture what can be conceptualized as an “adiposity-amplified insulin resistance phenotype”, where the degree of insulin resistance is proportionally weighted by the adiposity burden that drives and perpetuates the metabolic dysfunction (Lee et al., 2023; Kuang et al., 2023). The clinical utility of this approach has been extensively validated through multiple large-scale studies. Er et al. first demonstrated that TyG-BMI showed superior predictive capacity for insulin resistance compared to TyG alone, establishing it as a simple and clinically useful surrogate marker for insulin resistance in nondiabetic individuals (Er et al., 2016). Building on this foundation, subsequent cardiovascular outcome investigations confirmed that TyG-BMI provides significantly enhanced discrimination, with studies consistently showing that modified TyG indices outperform the traditional TyG index in predicting insulin resistance by accounting for body fat distribution and obesity’s contributory role (Ramírez-Vélez et al., 2019; Li et al., 2024). Recent prospective research by Xuan et al. demonstrated the superior performance of TyG-WHtR over TyG, WHtR, TyG-WC and TyG-BMI in predicting diabetes development (Xuan et al., 2022), while Park et al. further validated that TyG-BMI, TyG-WC, and TyG-WHtR were associated with increased risk of new-onset cardiovascular disease in participants without diabetes (Park et al., 2024). The prominence of TyG-BMI in our SHAP analysis actually validates rather than contradicts this comprehensive assessment strategy. Unlike standalone BMI, which provides only static anthropometric information, TyG-BMI represents a composite biomarker that integrates metabolic functionality (insulin resistance as reflected by triglyceride-glucose interaction) with adiposity burden (BMI). This combination captures the dynamic interplay between metabolic dysfunction and body composition that characterizes high-risk OSA-hypertension phenotypes. Mechanistically, our study demonstrates that TyG-BMI functions as a “metabolic amplifier” in OSA-hypertension patients, where the combination of insulin resistance and adiposity creates exponentially increased cardiovascular risk rather than simply additive effects. The superior predictive performance of TyG-BMI over isolated BMI in our models reflects this synergistic relationship: OSA-induced intermittent hypoxia simultaneously exacerbates insulin resistance (captured by elevated TyG values) and promotes visceral adiposity accumulation (reflected by BMI), creating a multiplicative cardiovascular risk profile that traditional anthropometric measures fail to capture. This mechanistic insight translates directly into clinical practice through our comprehensive risk stratification framework. TyG-BMI, integrated alongside our multi-dimensional feature set including sex, age, metabolic biomarkers and sleep parameters, enables identification of high-risk patient subgroups that would be missed by BMI-only assessment strategies. Patients with elevated TyG-BMI values identified by our predictive model represent a distinct high-risk phenotype requiring comprehensive metabolic assessment and intensive cardiovascular risk reduction strategies, moving beyond traditional BMI-based obesity management toward personalized metabolic risk stratification. This validates our study’s core premise: effective cardiovascular risk prediction in OSA-hypertension patients requires integrated evaluation of metabolic, anthropometric, demographic, and AHI rather than reliance on any single measurement, thereby supporting the superiority of our holistic machine learning-driven approach over traditional BMI-centric risk stratification methods.

Although obesity affects approximately one in eight individuals globally, no universal consensus has yet been reached regarding its classification and definition (Rubino et al., 2025). Moreover, the incidence of OSA in people with obesity can be as high as 30% (Rubino et al., 2025). OSA is a prevalent sleep disorder, wherein patients experience intermittent hypoxemia and sleep disruption due to recurrent upper airway obstructions during sleep (Yeghiazarians et al., 2021). Hypoxemia may lead to excessive activation of the sympathetic nervous system, thereby elevating blood pressure and impairing blood pressure control (He et al., 2020). Concurrently, fragmented sleep can induce hormonal imbalances, further exacerbating insulin resistance (IR) and metabolic disorders (Yeghiazarians et al., 2021). While multidimensional data offer a more comprehensive insight into patient status, information overload may complicate clinical decision-making. Currently, machine learning techniques can effectively elucidate the relationships between potential risk factors and prognosis by leveraging multidimensional data (Krittanawong et al., 2017; Wang et al., 2024a; Wang et al., 2024b; Yao et al., 2024; Zhu et al., 2025; Zhang and Li, 2024). This approach not only enhances the precision of risk assessment but also enables the visualization of associations between individual variables and long-term outcomes. By applying machine learning methods, we can identify critical determinants of adverse long-term outcomes and quantify their impact with greater accuracy (Wang J. et al., 2025; Krittanawong et al., 2017; Wang et al., 2024a; Wang et al., 2024b; Yao et al., 2024; Zhu et al., 2025; Wang Y. et al., 2025). Such visualization aids in a deeper understanding of the roles of various variables, thereby assisting clinicians in developing more personalized treatment plans that account for each patient’s unique circumstances. Furthermore, machine learning can uncover complex relationships that traditional statistical methods may overlook, providing a more robust foundation for clinical decision-making (Wang J. et al., 2025; Krittanawong et al., 2017; Wang et al., 2024a; Wang et al., 2024b; Yao et al., 2024; Zhu et al., 2025; Wang Y. et al., 2025). To more effectively evaluate cardiovascular disease risk and address obesity in patients with OSA and hypertension, our study employs various machine learning techniques to elucidate the relationships between potential risk factors and prognosis. Our model enhances the precision of risk assessment and enables the visualization of associations between individual variables and long-term outcomes.

Notably, obesity is recognized as a chronic, systemic disease characterized by excessive fat accumulation (Rubino et al., 2025). This new perspective underscores the intricate and multifaceted nature of obesity, highlighting its profound impact on individual health. It involves a complex interplay of various determinants that collectively contribute to its development (Rubino et al., 2025). In our study, leveraging advanced machine learning technologies, we can more accurately analyze and predict health risks associated with obesity and metabolic status, identify potential comorbidities, and develop personalized intervention strategies. Based on this comprehensive approach, we aim to develop novel and evidence-based strategies and solutions for the prevention and management of obesity. In clinical practice, these indices are easy to calculate and reproducible and can be obtained from routine physical examination data. Regular monitoring of these indices facilitates the early identification of high-risk patients, enabling timely personalized interventions. Moreover, our data provide a more nuanced understanding of obesity than the current definition, emphasizing the significance of identifying subtle differences between individuals and guiding more precise treatment for patients having OSA with hypertension.

### Study limitations

First, as a retrospective study, there is a risk of selection bias and unmeasured confounders. As the baseline data were derived from electronic health record, there may have been missing information or inaccurate records. Second, the absence of advanced imaging-based omics approaches, particularly radiomics and imaging genomics, restricts our capacity to comprehensively characterize the phenotypic heterogeneity of obesity. Imaging omics techniques, such as the quantitative analysis of adipose tissue distribution, composition, and metabolic activity using advanced imaging modalities, could provide deeper insights into the morphological and functional aspects of obesity beyond traditional anthropometric measurements. Finally, although the sample size was adequate for the primary analysis, it may be insufficient when performing a subgroup analysis, particularly when evaluating the specific effects of different modified TyG indices on different populations.

## Conclusion

We developed a novel predictive model that integrates diverse machine learning algorithms with multidimensional datasets to identify individuals at high risk of obesity-associated long-term MACCEs. Furthermore, we redefined the concept of obesity through a TyG index that incorporates body shape and fat distribution parameters, thereby enabling a more comprehensive assessment of insulin resistance and cardiovascular risk stratification.

## Data Availability

Individual participant data that underlie the results reported in this article, after de-identification can be obtained from the corresponding author upon reasonable request.
