# Supplementary material for: Multiple machine-learning-driven metabolic frameworks for long-term prognostic risk assessment in patients with coexisting hypertension and obstructive sleep apnea:insights from a multicenter cohort study
Source: Front Physiol. 2026 Feb 24;17:1739374. doi: 10.3389/fphys.2026.1739374 (PMC12971643; doi:10.3389/fphys.2026.1739374)

**Supplemental** **[Table 1](https://www.frontiersin.org/articles/10.3389/fcvm.2022.888753/full#T2). Inclusion and exclusion criteria.**

| **Inclusion criteria** |
| --- |
| Obstructive sleep apnea with an Apnea-Hypopnea Index (AHI) of 5 or greater signifies the presence of sleep-disordered breathing events |
| Hypertension |
| **Exclusion criteria** |
| Acute coronary syndrome |
| Acute cardiac failure |
| Implantation of pacemaker |
| Acute- and chronic-phase inflammatory responses |
| Depressed left ventricular systolic function (ejection fraction <50%) |
| Malignant tumor |
| Severe aortic stenosis |
| Dilated cardiomyopathy |
| Rheumatic heart disease |
| Cor pulmonale |
| Myocarditis or cardiomyopathy |
| Infectious or severe liver or kidney disease |
| Lacked data on fasting triglyceride and fastingblood-glucose |
| Patients without the results of a sleep monitoring study |
| Poor compliance to treatment (Patients who neglected antihypertensive medication adherence [defined as medication adherence <80% based on pill counts, pharmacy refill records, or self-reported non-adherence during structured interviews] or patients who did not attend outpatient follow-up visits (defined as missed scheduled appointments >30% over a 12-month period or complete absence from scheduled follow-up for >6 consecutive months). |
| Lost to follow-up |

**Supplemental Table 2. Variance inflation factor .**

|  | ***Variance inflation factor*** |
| --- | --- |
|  |  |
| Creatinine | 1.089 |
| Fibrinogen | 1.109 |
| AHI | 1.259 |
| Male sex | 1.334 |
| Age | 1.389 |
| TyG-BMI | 1.447 |
| HbA1c | 1.865 |
| Fasting plasma glucose | 2.730 |
| Triglyceride | 3.806 |
| TyG | 5.211 |

**Supplemental Table 3. Optimal parameters of nine machine learning models in predicting MACCEs rates.**

| **Machine learning models** | **Optimal parameter** |
| --- | --- |
|  |  |
| XGBoost | Reg Lambda (L2 Regularization Coefficient): 0.5  Min Child Weight: 2  Max Depth: 4  Learning Rate: 0.3 |
| LightGBM | Num Leaves (Maximum Number of Leaves): 5  N Estimators (Maximum Number of Trees): 5  Max Depth: 1  Learning Rate: 0.001  Boosting Type: DART |
| Random Forest | N Estimators (Number of Trees): 100  Min Impurity Decrease: 0.0  Max Depth: None  Criterion: Gini |
| Decision Tree | Min Samples Split: 50  Min Samples Leaf: 1  Max Depth: 1  Criterion: Gini |
| Gradient Boosting | N Estimators (Maximum Number of Trees): 100  Min Samples Split: 50  Min Samples Leaf: 50  Max Depth: 20  Loss: Exponential  Learning Rate: 2 |
| Multi-Layer Perceptron | Max Iterations: 10  Hidden Layer Sizes: (10, 10)  Activation Function: Logistic |
| Support Vector Machine | Tolerance (Tol): 0.001  Kernel Type: RBF  Regularization Factor (C): 1.0 |
| K-Nearest Neighbors | Weights: Uniform  Number of Neighbors (n_neighbors): 2 |
| Gaussian Naive Bayes | Variance Smoothing (var_smoothing): 1e-07 |

**Supplemental Table 3. Performance of nine machine learning models in training cohort.**

| Training cohort | AUC (95%CI) | Accuracy | Sensitivity | Specificity | Positive predictive value | Negative predictive value | F1-score | Kappa |
| --- | --- | --- | --- | --- | --- | --- | --- | --- |
| XGBoost | 0.911 (0.882-0.940) | 0.859 | 0.778 | 0.876 | 0.57 | 0.949 | 0.658 | 0.572 |
| LightGBM | 0.911 (0.887-0.936) | 0.846 | 0.848 | 0.846 | 0.538 | 0.963 | 0.659 | 0.566 |
| Random Forest | 1.000 | 1.0 | 1.0 | 1.0 | 1.0 | 1.0 | 1.0 | 1.0 |
| Decision Tree | 0.629 (0.584-0.675) | 0.848 | 0.293 | 0.966 | 0.644 | 0.866 | 0.403 | 0.329 |
| Gradient Boosting | 0.629 (0.584-0.675) | 0.848 | 0.293 | 0.966 | 0.644 | 0.866 | 0.403 | 0.329 |
| Multi-Layer Perceptron | 0.654 (0.595-0.713) | 0.647 | 0.586 | 0.66 | 0.267 | 0.883 | 0.367 | 0.167 |
| Support Vector Machine | 0.826 (0.781-0.870) | 0.701 | 0.859 | 0.668 | 0.354 | 0.957 | 0.501 | 0.337 |
| K-Nearest Neighbors | 0.914 (0.893-0.936) | 0.731 | 1.0 | 0.675 | 0.394 | 1.0 | 0.566 | 0.42 |
| Gaussian Naive Bayes | 0.820 (0.776-0.864) | 0.708 | 0.828 | 0.683 | 0.357 | 0.949 | 0.498 | 0.336 |

**Supplemental Table 4. Performance of nine machine learning models in validation cohort.**

| Validation cohort | AUC (95%CI) | Accuracy | Sensitivity | Specificity | Positive predictive value | Negative predictive value | F1-score | Kappa |
| --- | --- | --- | --- | --- | --- | --- | --- | --- |
| XGBoost | 0.898 (0.822-0.973) | 0.887 | 0.741 | 0.922 | 0.69 | 0.938 | 0.714 | 0.644 |
| LightGBM | 0.813 (0.704-0.923) | 0.817 | 0.704 | 0.843 | 0.514 | 0.924 | 0.594 | 0.479 |
| Random Forest | 0.889 (0.800-0.977) | 0.873 | 0.37 | 0.991 | 0.909 | 0.87 | 0.526 | 0.468 |
| Decision Tree | 0.654 (0.562-0.745) | 0.852 | 0.333 | 0.974 | 0.75 | 0.862 | 0.462 | 0.39 |
| Gradient Boosting | 0.654 (0.562-0.745) | 0.852 | 0.333 | 0.974 | 0.75 | 0.862 | 0.462 | 0.39 |
| Multi-Layer Perceptron | 0.630 (0.512-0.748) | 0.599 | 0.593 | 0.6 | 0.258 | 0.863 | 0.36 | 0.129 |
| Support Vector Machine | 0.860 (0.768-0.952) | 0.697 | 0.889 | 0.652 | 0.375 | 0.962 | 0.527 | 0.355 |
| K-Nearest Neighbors | 0.677 (0.558-0.796) | 0.585 | 0.667 | 0.565 | 0.265 | 0.878 | 0.379 | 0.147 |
| Gaussian Naive Bayes | 0.831 (0.727-0.935) | 0.711 | 0.852 | 0.678 | 0.383 | 0.951 | 0.529 | 0.361 |

**Supplemental Figure 1. ROC curves were used to evaluate the predictive accuracy and discriminative power of the Cox proportional hazards regression model utilizing backward stepwise selection in training (A) and independent validation (B) cohorts.**


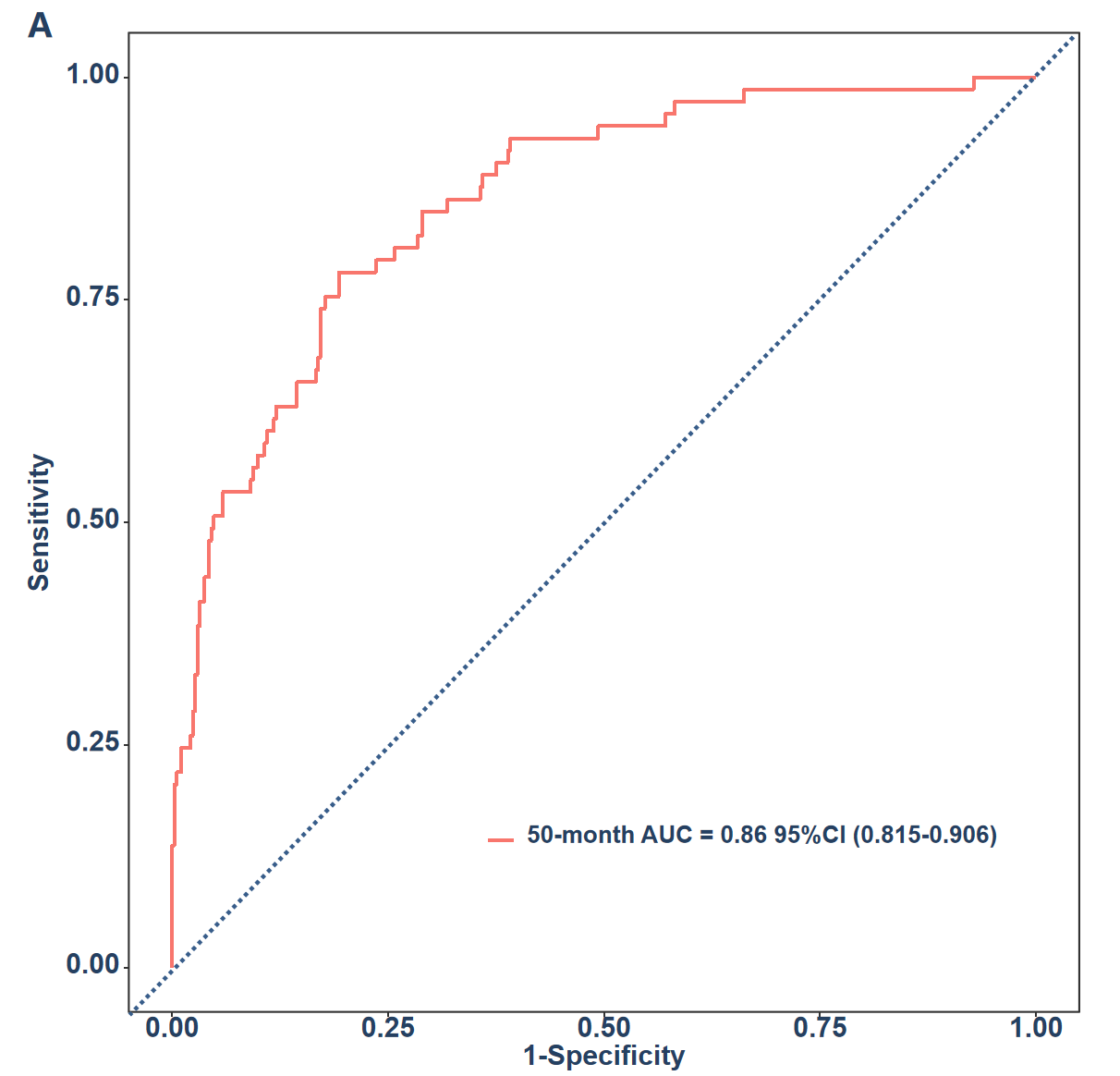


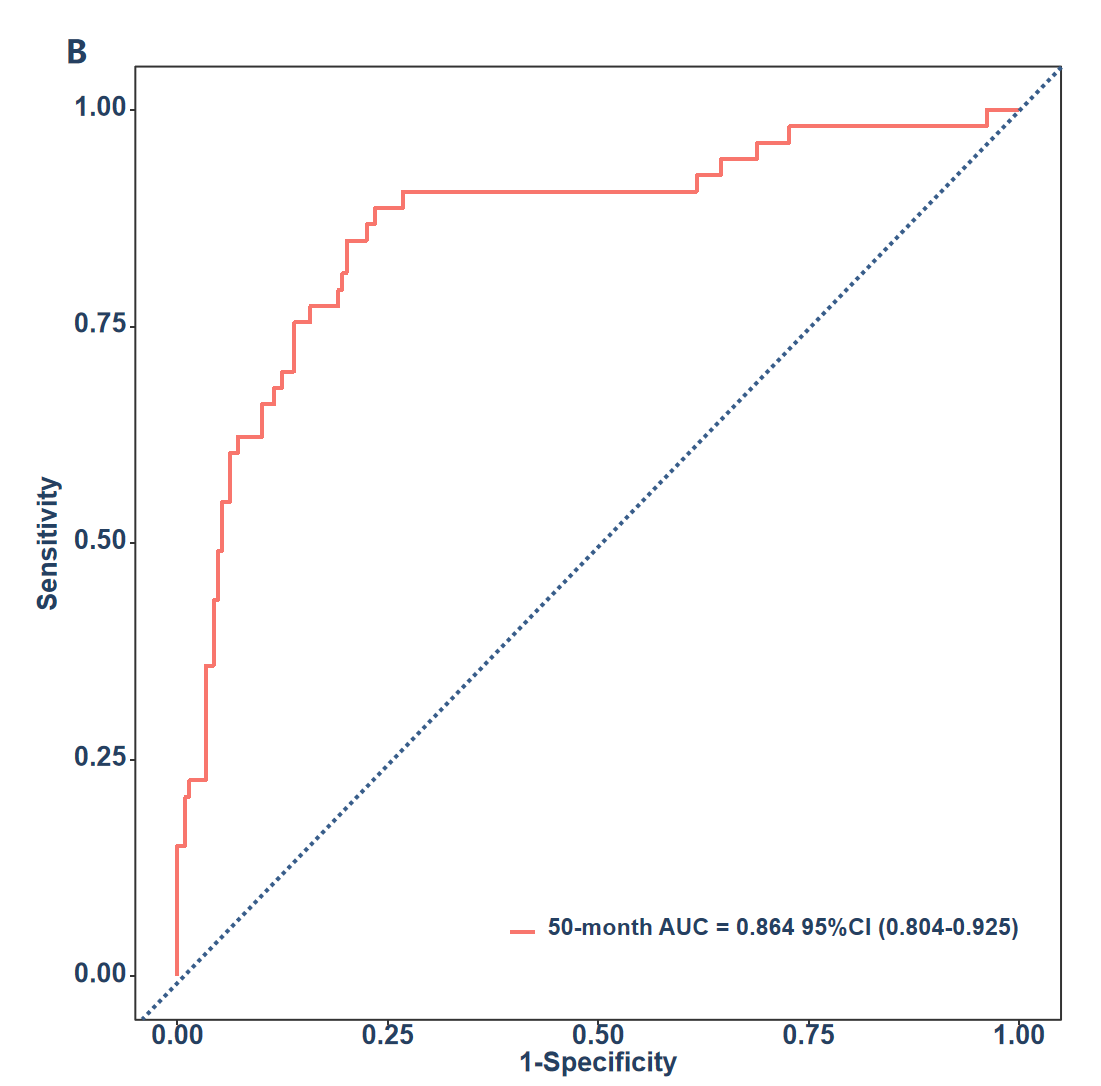


**Supplemental Figure 2. The importance score ranking of the XGBoost model is depicted through the mean absolute SHAP (SHapley Additive exPlanations) values associated with various clinical and metabolic features, thereby highlighting their average impact on the model's output magnitude. The y-axis enumerates the features in descending order of importance, while the x-axis denotes the mean absolute SHAP value, quantifying the contribution of each feature to the model's predictions.**


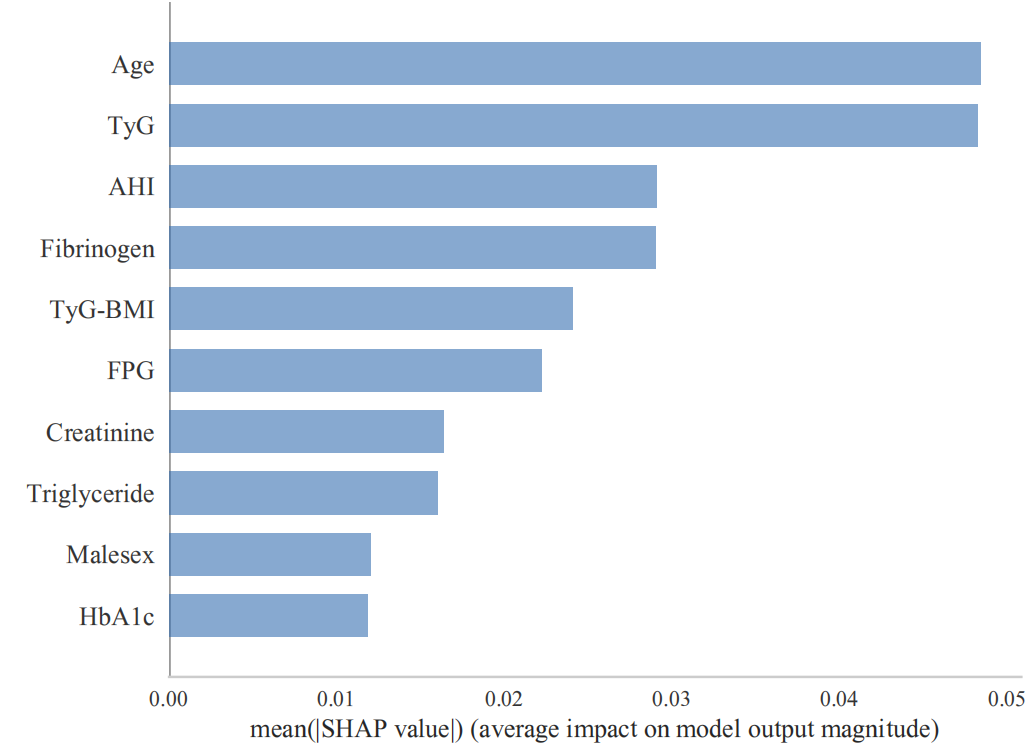


**Supplemental Figure 3. The figure consists of two scatter plots (A and B) that illustrate the relationship between SHAP (SHapley Additive exPlanations) values and two metabolic predictors: the Triglyceride-Glucose (TyG) index and the TyG-Body Mass Index (TyG-BMI).**


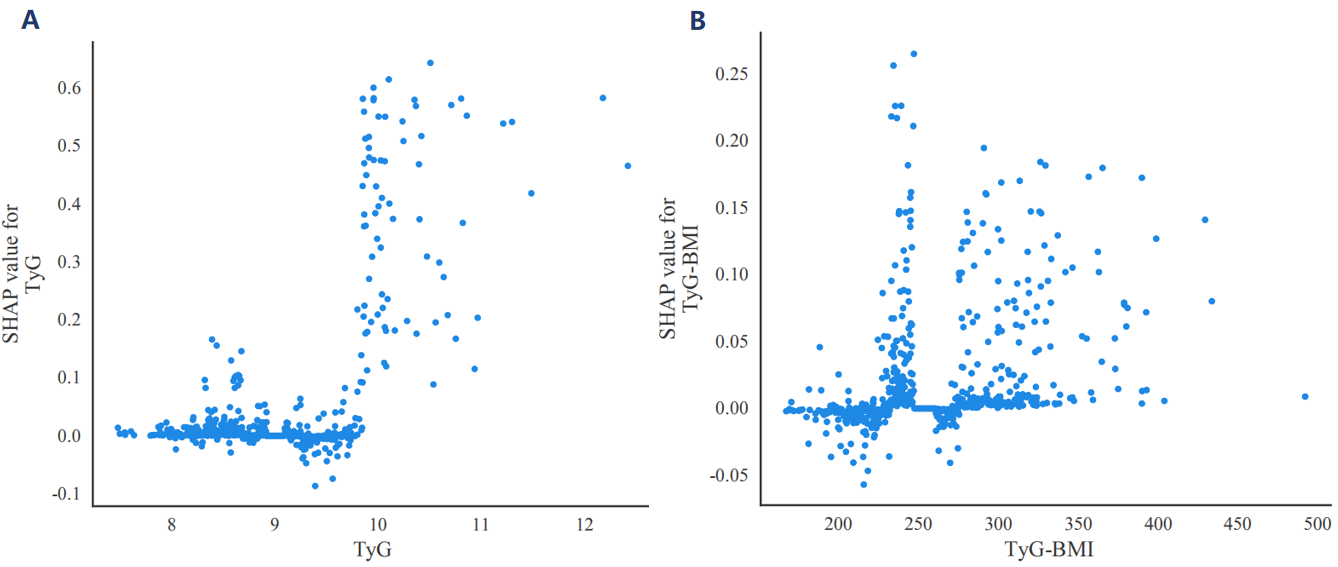

Supplement: Supplementary file 1 [file DataSheet1.docx]
